# Early Diagnosis of Knee Osteoarthritis With a Natural Language Processing–Driven Approach Based on Clinician Notes: Development and Validation Study

**DOI:** 10.2196/64536

**Published:** 2025-08-14

**Authors:** Narathip Thanyakunsajja, Kulsawasd Jitkajornwanich, Shan Xu, Donghee Shin, Pattama Charoenporn

**Affiliations:** 1 King Mongkut’s Institute of Technology Ladkrabang Bangkok Thailand; 2 Texas Tech University Lubbock, TX United States

**Keywords:** computational communication, health informatics, natural language processing, artificial intelligence, text mining

## Abstract

**Background:**

Knee osteoarthritis (OA) is a common form of knee arthritis that can cause significant disability and affect a patient’s quality of life. Although this disease is chronic and irreversible, the patient’s condition can be improved and the progression of the disease can be prevented if the disease is diagnosed early and the patient receives appropriate treatment immediately. Therefore, the prediction of knee OA is considered one of the essential steps to effectively diagnose and prevent further severe OA conditions. Knee OA is commonly diagnosed by medical experts or physicians, and the diagnosis of OA is mainly based on patients’ laboratory results and medical images, including x-ray and magnetic resonance images. However, diagnosis through such data is often time-consuming. Moreover, the diagnosis results can vary among physicians depending on their expertise. Previous studies mostly focused on using approaches, such as those involving artificial intelligence, to automatically detect knee OA through such data. However, these studies did not incorporate clinicians’ or doctors’ notes (text data) into the analysis, although these data involving reported symptoms and behaviors are already available and easier to collect and access than laboratory data and image data.

**Objective:**

We propose a novel natural language processing–driven approach based on clinicians’ or doctors’ notes of patient-reported symptoms (text data only) for diagnosing knee OA.

**Methods:**

The textual information from clinicians’ or doctors’ notes was first preprocessed using text analysis algorithms with respect to natural language processing. We then incorporated deep learning models, including convolutional neural networks, bidirectional long short-term memory (BiLSTM), and gated recurrent units. Lastly, a disease-specific standard questionnaire called WOMAC (Western Ontario and McMaster Universities Arthritis Index) was taken into account to improve the overall performance of the models.

**Results:**

Our experiment included 5849 records (OA: 3455; non-OA: 2394). Before applying our WOMAC-based processing approach, the best-performing model was BiLSTM (area under the curve, 0.85; accuracy, 0.87; precision, 0.85; sensitivity, 0.95; specificity, 0.76; *F*_1_-score, 0.90), and there was an improvement in the results with BiLSTM after applying our approach (area under the curve, 0.91; accuracy, 0.91; precision, 0.91; sensitivity, 0.94; specificity, 0.87; *F*_1_-score, 0.93).

**Conclusions:**

Our proposed method for predicting the occurrence of knee OA showed better performance than other conventional methods that use image data and statistical laboratory data. The findings indicate the feasibility of using text data (symptom descriptions reported by patients and recorded by doctors) to predict knee OA. Medical notes of symptom reports can be considered a valuable data source for predicting whether a particular knee is likely to experience OA progression.

## Introduction

### Background

Knee osteoarthritis (OA) is a long-term condition, and its cause is still unknown. The most common form of arthritis involves cartilage degradation or bone abnormalities [1-3]. Joint pain, stiffness, and limitation of knee movement are some of the primary symptoms of knee OA. Knee OA is a slow but aggressive disease, and as the damage to the cartilage and bones cannot be restored, a prevention protocol could be beneficial [4]. Knee OA affects 10%-15% of adults worldwide. In 2015, the World Health Organization (WHO) estimated that 9.6% of men and 18% of women aged 60 years or older had symptomatic knee OA. Knee OA symptoms affect mobility in 80% of cases, while 25% of people with symptomatic knee OA struggle to perform their daily activities. Knee OA can not only affect individuals both physically and psychologically but also create a burden on society and the economy [5]. In 2008, knee OA was ranked as one of the most expensive diseases to treat. The cost of hospital treatment for arthroplasty surgery has reached approximately US $40 billion worldwide [6]. Having said that, knee OA can be diagnosed at an early stage of the disease. Early detection and diagnosis are possible and crucial to overcome the issues associated with this highly disabling disease, as early-stage treatment can prevent further breakdown of the bone and cartilage [1,7].

Owing to the presence of big data and the advancement of artificial intelligence (AI), many researchers are using these to identify key factors influencing health and disease-related issues with the help of machine learning models. Furthermore, deep learning is one of the new and rising trends and opportunities in the field of machine learning for many studies in diverse domains. This is particularly true in the medical domain, where deep learning and natural language processing (NLP) could be used for medical text classification tasks [8,9]. This has led to the emergence of new research areas in medical text classification and summarization. Machine learning approaches for text classification can be roughly divided into 2 types: traditional machine learning approaches and deep learning–based approaches. Because traditional approaches have a limited ability to process large text data, which requires complex computations in the corpus, the performance of models is greatly affected. On the other hand, deep learning models have been shown to be highly effective in text classification [10]. Moreover, many deep learning models, such as recurrent neural networks (RNNs), long short-term memory (LSTM), convolutional neural networks (CNNs), and gated recurrent units (GRUs), are widely used in the field of text classification [11,12]. To use deep learning in NLP, we need a form of word representation to transform words into vectors, known as word embeddings. One of the most widely used methods for this is Word2Vec [13,14], which we adopted in our work.

In this study, we have proposed an NLP model to detect the occurrence of knee OA by incorporating deep learning models (GRUs, bidirectional long short-term memory [BiLSTM], and CNNs) based on the textual data of knee OA symptoms reported in doctors’ notes. Moreover, we have performed a literature review. The methodology of the proposed sentiment analysis has been explained in the Methods section, and the results have been provided in the Results section. Finally, discussions, conclusions, and future work have been communicated in the Discussion section.

### Literature Review

In the medical AI–related literature, deep learning has been used to help detect and diagnose diseases. Often, each study is differentiated by the type of data used, such as images and statistical data, including laboratory results. We have discussed several studies in this section related to the use of AI, machine learning, and deep learning for detecting and predicting knee OA based on both medical images and statistical data, including laboratory results.

In the study by Brahim et al [1], x-ray images of the knees of patients, together with machine learning algorithms, were used to develop a computer-aided diagnosis (CAD) system for detecting early knee OA. The machine learning algorithms used were naïve Bayes and random forest models, which were adopted for classification tasks. The approach used 1025 knee x-ray images from the public Knee Osteoarthritis Initiative database [15,16]. Yong et al [17] proposed a method to detect Kellgren/Lawrence (KL) grading [18], using an ordinal regression module (ORM) in neural networks. The module can predict the inclusion of images in multiple classes that follow a natural severity order, ranging from KL grades 0 to 4. They also used data from the Knee Osteoarthritis Initiative [15,16]. A total of 4130 pairs of knee images were labeled with KL grades and collected from the baseline cohort. These studies yielded good results, but they only considered x-ray images (data were obtained from x-ray machines). Halilaj et al [19] proposed a mixed effects model to differentiate clusters of knee OA progression. They used data from 1234 patients in the Knee Osteoarthritis Initiative, who were classified as being at high risk of developing knee OA based on age, BMI, and medical and occupational histories, to identify knee OA progression. They also developed models to predict the probability of belonging to a cluster using Least Absolute Shrinkage and Selection Operator (LASSO) regression. Lim et al [6] proposed a feed-forward neural network trained with standard back propagation as a deep learning model to detect the occurrence of knee OA using the statistics of patients, including medical and health-related behavioral information. They used data from 5749 subjects in the Korea National Health and Nutrition Examination Survey [20], which was performed by the Korea Centers for Disease Control and Prevention (KCDC). Wang et al [21] developed a model to assess the severity of radiographic knee OA and identify key risk factors for early intervention and treatment. To predict the KL grade, they used a LSTM model with an attention mechanism based on data from 518 patients in the Knee Osteoarthritis Initiative [15,16]. From these previous studies, we noticed that even though the statistical methods were faster than the image methods, the high accuracy of the image methods yielded better results compared to the statistical methods. Recently, Tiulpin et al [22] developed a machine learning approach to predict the progression of structural knee OA, using raw radiographic image data, physical examination results, patient medical history, anthropometric information, and KL grade as features. They included data from the Knee Osteoarthritis Initiative (2711 subjects) [15,16] in the training set and data from the Multicenter Osteoarthritis Study (2129 subjects) [16,23] in the test set. The experiments combined both the image method and statistical method and yielded good performance. However, this approach considered x-ray images, which need to be retrieved from x-ray machines and take a long time to process.

Recently, the field of NLP has shown significant technological advances, especially in text classification involving assigning a label or class to a given text. Text classification has many practical applications, such as spam detection in email servers, news categorization, sentiment analysis, hate speech detection in social media, etc. It has also been applied to medical fields. For example, Liu et al [8] applied text classification to label words retrieved from social media big data as medical terms and categorized them accordingly.

Traditional text classification approaches are dictionary-based and involve basic machine learning methods [24]. Differentiating texts related to patients is challenging. This is because the texts used in the medical field are still unstructured data and can be complicated to process, especially when processed by basic machine learning approaches [25]. On the other hand, deep learning methods have shown strong performance in image classification as well as speech recognition. These methods have been widely used in the fields of NLP and text classification and have yielded good results [26]. CNNs and RNNs are among the most widely adopted deep learning models for text classification.

Jang et al [24] proposed a method to classify movie user reviews into positive, negative, and neutral categories using a sentiment analysis model that combines BiLSTM and CNN. The Internet Movie Database (IMDb) movie review dataset was used for this experiment. The model used a CNN to reduce the number of input features. The features extracted from the CNN were input into a BiLSTM model for the final classification. Qing et al [26] proposed a neural network approach to classify medical texts into one of four dataset categories: (1) clinical records from Traditional Chinese Medicine in the Classified Medical Records of Distinguished Physicians Continued Two (TCM), (2) an open inpatient medical records dataset from the China Conference on Knowledge Graph and Semantic Computing (CCKS), (3) a corpus of biomedical publication abstracts labeled for cancer hallmarks, and (4) a dataset of Activating Invasion and Metastasis (AIM). They employed a convolutional layer to extract features from sentences and used a bidirectional gated recurrent unit (Bi-GRU) for classification.

Many previous studies have focused on image and statistical data for knee OA prediction with machine learning and deep learning techniques. However, a limitation of image data is that they must be generated and retrieved through x-ray machines or magnetic resonance imaging (MRI) scanners. Moreover, statistical data have limitations associated with the integrity of the factors and variables related to patients.

### Objectives

NLP or text classification has potential and could be beneficial in knee OA prediction. For the early detection of knee OA, we propose a novel approach based on an NLP or text classification method that uses only textual data, instead of x-ray or MRI image data and statistical data, and incorporates deep learning models, including GRU, BiLSTM, and CNN models. We used textual data in our research, which included clinicians’ or doctors’ notes on patient symptoms related to knee OA.

## Methods

### Ethical Considerations

Data permission was approved and granted by the study hospital and the associated institutions according to the institutional review board (IRB2023-736; develop language models for OA diagnosis). Regarding our dataset, no identifiable data of patients were collected in this study. The CONSORT (Consolidated Standards of Reporting Trials) (Multimedia Appendix 1), CONSORT-EHEALTH (Consolidated Standards of Reporting Trials of Electronic and Mobile Health Applications and Online Telehealth) (Multimedia Appendix 2), and STARD (Standards for Reporting of Diagnostic Accuracy Studies) (Multimedia Appendix 3) checklists [27-29] were followed, and training was provided in human ethics for conducting research in humans.

### Resources

All models were trained using a MacBook Pro 2018 laptop having an Intel Core i5 quad-core CPU (2.3 GHz), an Intel Iris Plus 655 GPU, and a Macintosh disk storage (256 GB).

### Study Design and Data Sources

The approach comprised 6 stages. First, deidentified data on patients’ reported symptoms from clinicians’ or doctors’ notes were obtained from a hospital in Thailand (N=6737; knee OA: 3945, non-OA: 2792). No personal attributes or biospecimen data, such as laboratory results, were used. Second, data selection, filtering, cleaning, and preparation processes were performed for the data of patients who met the criteria of the experiments (N=5849; knee OA: 3455, non-OA: 2394). Third, text analysis and mining processes were performed on the datasets. Fourth, to enhance the efficiency of the model, we incorporated our text analysis and mining models with the WOMAC (Western Ontario and McMaster Universities Osteoarthritis Index) and specific concepts in our AI model. Fifth, to conform to reporting guidelines, we randomized our data into 3 groups: training, validation, and test sets. Finally, we applied and conducted comparison experiments with deep learning models (ie, CNNs, BiLSTM, and GRUs) to predict knee OA diagnosis.

Texts in the medical field mainly contain 3 types of words. The first category is standardized medical terminology, which refers to specific concepts in the medical field. The second category is user-based health terminology, such as “can’t sleep” and “stomach pain.” The third category includes words that are considered noise, such as “because” and “so.” These words typically lack relevant health information and can cause interference [8]. Our dataset primarily combined the first 2 categories. However, the data in this work consisted of words describing symptoms, which can be varied (such as pain [“
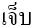
” in Thai] and ache [“
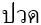
” in Thai], where both words mean “pain”). Furthermore, the data may have some incorrect words in sentences. Therefore, one of the core steps in our methodology was to use a WOMAC-based processing approach to transform some words in sentences that are similar in the WOMAC corpus to medical terminology by applying the *SequenceMatcher* library [30]. We defined a cutoff score of similarity of 0.85 to replace old words with WOMAC words.

**Figure 1:**
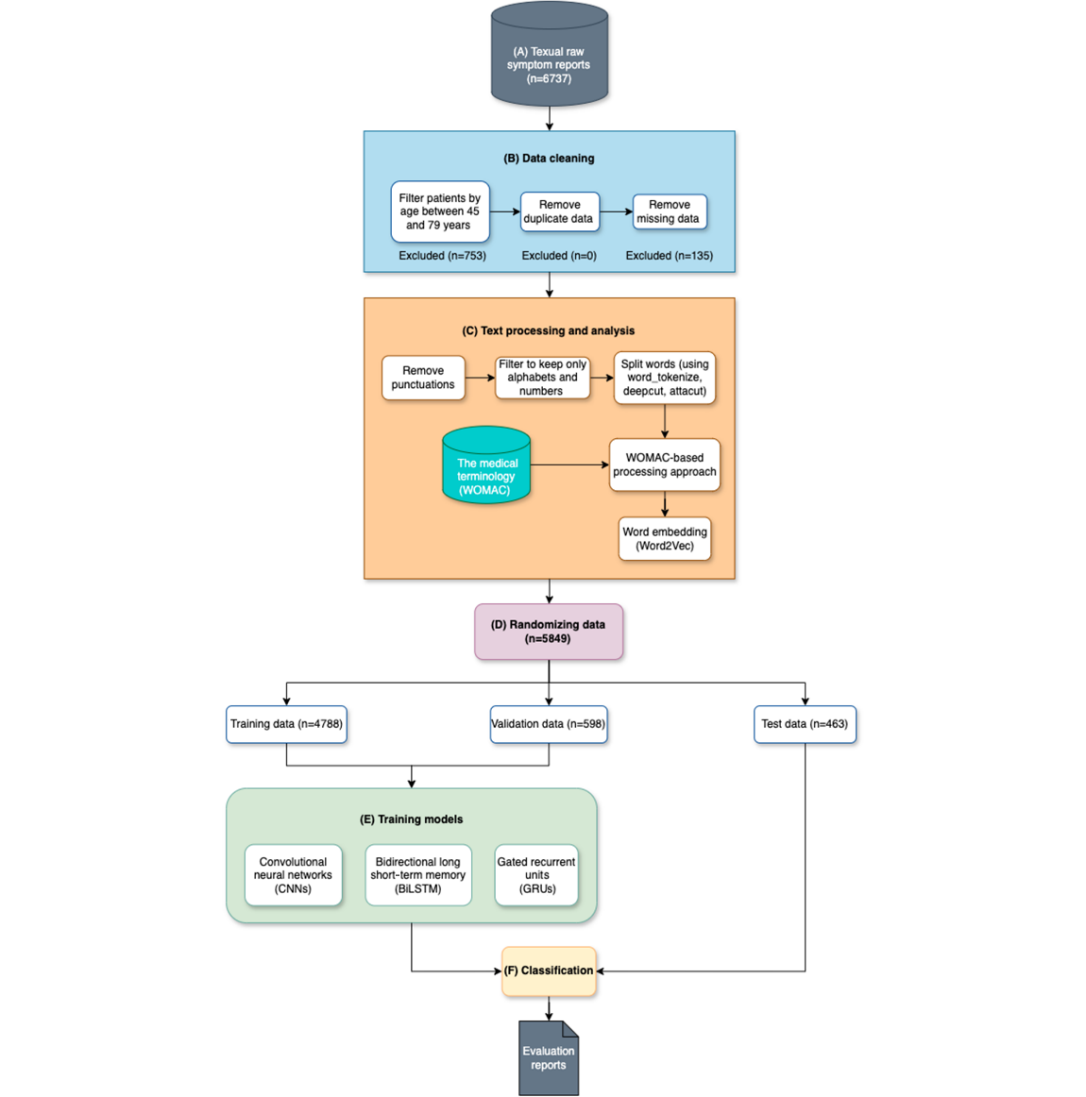
Schematic representation of our knee osteoarthritis prediction model, consisting of (A) textual raw symptom reports, (B) data cleaning, (C) text processing and analysis, (D) randomizing data (with respect to CONSORT [Consolidated Standards of Reporting Trials], CONSORT-EHEALTH [Consolidated Standards of Reporting Trials of Electronic and Mobile Health Applications and Online Telehealth], and STARD [Standards for Reporting of Diagnostic Accuracy Studies] checklists), (E) training models, and (F) classification. WOMAC: Western Ontario and McMaster Universities Osteoarthritis Index.

To conform to appropriate reporting guidelines, CONSORT 2010 [27] and CONSORT-EHEALTH [28] guidelines were applied in randomizing our data into 3 groups: training, validation, and test sets. Moreover, STARD 2015 [29] guidelines were used to ensure the quality and clarity of reporting of our diagnostic accuracy studies. Figure 1 illustrates the process in detail.

### Data Preparation and Experiment Plan

#### Data Collection

The text reports of knee OA were provided by a hospital in Thailand and were collected by orthopedists between 2019 and 2021. Specifically, we focused only on textual knee OA reports on symptoms in the Thai language, and we considered only data from patients who first visited the hospital. To conduct fair experiments, we only considered patients who were new and had not been treated by orthopedists before, as we focused on patients who had not received treatment by orthopedists. We did not consider patients who had been treated previously, as their symptoms may not be clearly mentioned in their reports, which can degrade the accuracy of our model and predictions. The medical terminology (from the WOMAC corpus) was downloaded from the Royal College of Physiatrists of Thailand [31].

#### Data Cleaning

The data cleaning process involved selection, cleaning, filtering, and preparation. After obtaining medical text reports from the hospital, we investigated the data and excluded patient data that were incomplete or duplicate in order to keep only complete patient data. Patients aged between 45 and 79 years were considered for 2 reasons. First, many previous studies focused on data from patients aged between 45 and 79 years [1,17,19,21,22]. Second, in the Knee Osteoarthritis Initiative datasets [15,16], which are considered some of the standard resources to study the progression of OA and develop innovative approaches to treat this chronic disease, the focus is on patients aged 45-79 years.

#### Text Analysis and Processing

Data preprocessing was conducted. Punctuations were removed, and the texts were filtered to include only Thai alphabets and numbers. Moreover, word segmentation, tokenization, and correction of misspelled words were performed to ensure data quality. Next, we incorporated word embedding with the WOMAC and concepts. Each word was initially transformed into its embedding vector, with positional encoding and attention applied to assign weight to the words in each sentence. This yielded the final sentence vector.

#### Experiment Plan

We planned to use and experiment with 3 classification models, including CNN, BiLSTM, and GRU models, along with NLP components, which have been discussed in detail in the next section.

### NLP and Deep Learning Techniques

In this section, we describe the NLP components used in our research, namely, word segmentation and Word2Vec, as well as deep learning models.

#### Word Segmentation

Since Thai sentences can be verbose and lead to ambiguity, word segmentation was considered before conducting further analysis of the text. The 3 most prevalent word segmentation approaches for Thai sentences are presented below.

#### Word_Tokenize

Word_Tokenize [32,33] is one of the functions in the PyThaiNLP library. Word_Tokenize uses the maximal matching algorithm, which involves dictionary-based maximum matching and uses the Thai character cluster. Results demonstrated that it is the fastest word tokenizer on the BEST 2010 [34] benchmark, with 71.18% accuracy [35,36].

#### DeepCut

DeepCut [37] is a technique built on a CNN that uses characters and character types as features. The goal of the model is to predict whether a character is the beginning of a word before word segmentation can be processed. The BEST 2010 corpus [34] was used for training when building the model. Results showed that the model had 97.8% precision, 98.5% recall, and 98.1% *F*_1_-score [32].

#### AttaCut

Inspired by DeepCut, AttaCut [38] was designed to speed up the segmentation process. It achieves this goal by disabling neurons in DeepCut’s layers, and at the end, its speed performance increased by 6 times. Results on the in-domain dataset revealed 89%-91% *F*_1_-scores, and those on the out-domain dataset revealed 63%-81% *F*_1_-scores [32].

#### Word2Vec

**Figure 2:**
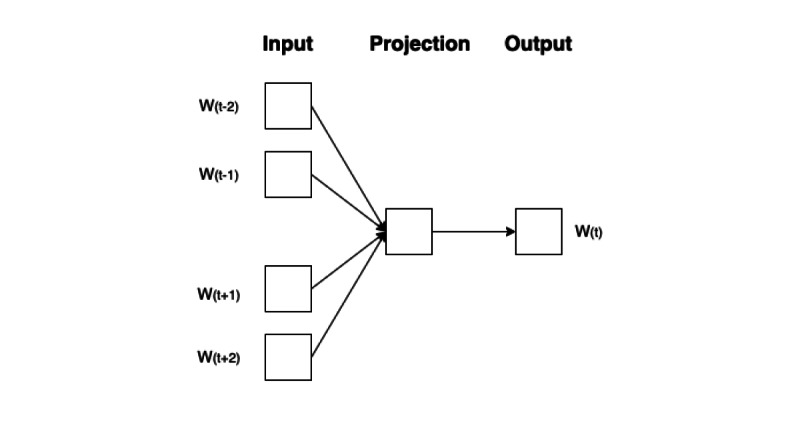
Continuous bag of words model architecture: one of the algorithms inside Word2Vec to produce the word vector of the current word by considering surrounding words.

**Figure 3:**
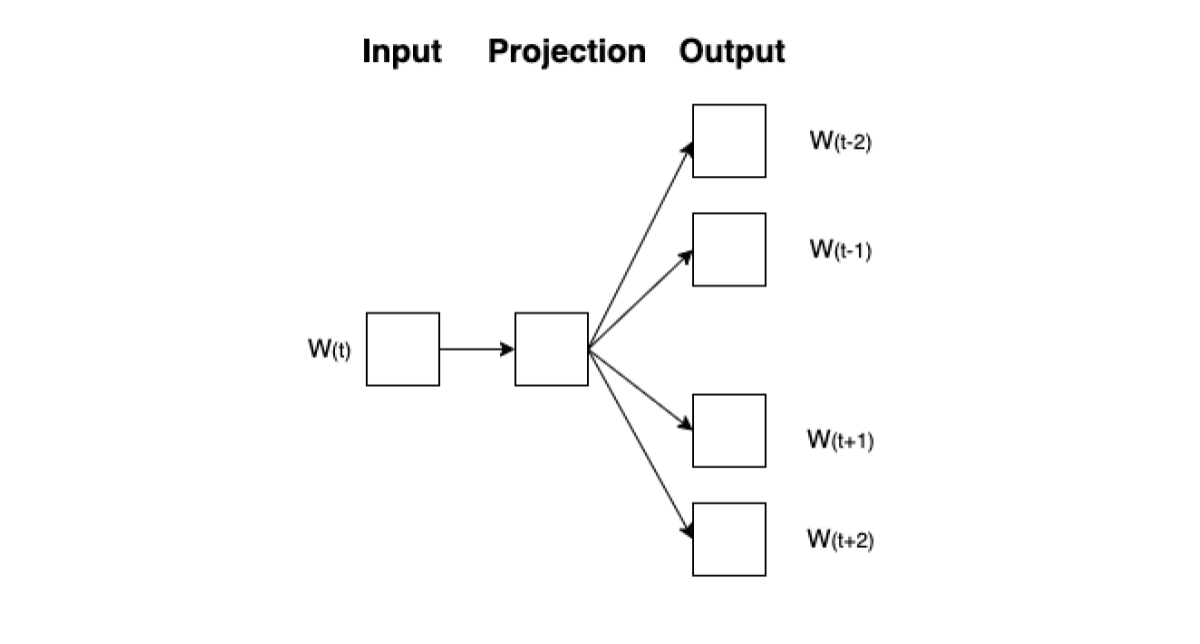
Skip-gram model architecture: one of the algorithms inside Word2Vec to produce word vectors for the surrounding words by considering the current word.

The well-known word embedding or word vector is an approach for representing documents and words. It is defined as a numeric vector input that allows words with similar meanings to have the same representation. Word2Vec is one of the models used to transform words and represent them as distributed vectors. It is commonly used as an initial step in predictive models for tasks related to semantics and information retrieval [24,39,40]. A neural network is then used to generate word embeddings as weights (by considering relationships between words and their surrounding context). Word2Vec generates word vectors using 2 main algorithms: continuous bag of words (CBOW) and skip-gram [41,42]. The CBOW and skip-gram architectures are shown in Figures 2 and 3, respectively.

Skip-gram is slower than CBOW in training and has lower performance on frequent words, but it is better suited for rare words or phrases. Since our data included medical terminology, which can contain rare words and phrases, we focused on Word2Vec by using skip-gram.

The skip-gram model generates word-context pairs (*w*, *c*) from text data as input for a neural network.

(*w*, *c*) = (*w*_1_, *c*_1_), (*w*_2_, *c*_2_), … , (*w_n_*, *c_n_*) **(1)**

*w_i_* ∈ *V_w_*, *c_i_* ∈ *V_c_*

where *V_w_* is the input vector (center word) and *V_c_* is the output vector (context word) that represent the word and context vocabularies with sizes *S_w_* and *S_c_*, respectively. The goal of the skip-gram model is to learn the distribution of each word (based on context) by maximizing the probability of context words, as shown below.



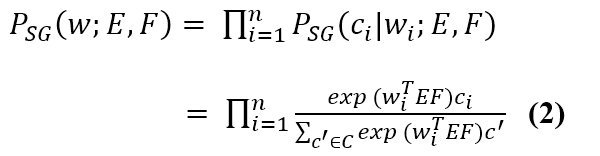



where *E* and *F* refer to the parameter matrices of (*S_w_*×*d*) and (*S_c_*×*d*), respectively, and *d* refers to the dimensionality of the embedding vector space.

#### Deep Learning Models

As mentioned in the literature review section, deep learning methods have demonstrated strong performance in image classification and speech recognition. In recent years, they have also been widely adopted in NLP and have achieved good performance as well [26]. In NLP tasks, the deep learning models widely used include GRU, LSTM, and CNN [11]. Therefore, we focused on these deep learning models for the detection of knee OA.

#### GRU Model

**Figure 4:**
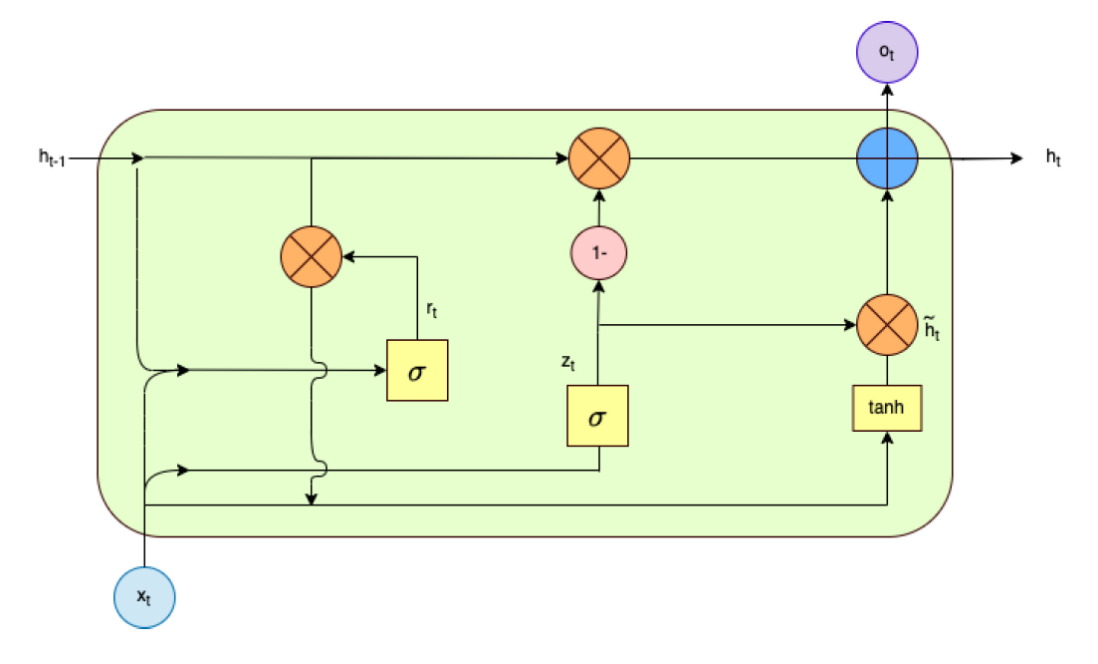
Gated recurrent unit model architecture: one of the recurrent neural network types. Its structure consists of input state (x_t_), hidden state (h_t_), output state (o_t_), and update gate (z_t_).

GRU is a type of RNN specifically designed to address the vanishing gradient problem when capturing long-term dependencies in RNNs. It is also very efficient in processing sequential or time series data in NLP tasks. The structure of GRU consists of an update gate and a reset gate [26,43]. GRU uses gating mechanisms to control which information should be passed to the output or remain in the network. GRU model architecture is illustrated in Figure 4.

The GRU structure is illustrated in Figure 4. In the equations listed below, the input *x_t_* (the sequence vector of the current word at time *t*) is the input for the hidden state *h_t_*.

*r_t_* = *σ*(*W_r_x_r_* + *U_r_h_t_*_-1_ + *b_r_*) **(3)**

*z_t_* = *σ*(*W_z_x_t_* + *U_z_h_t_*_-1_ + *b_z_*) **(4)**

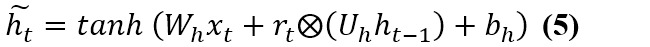





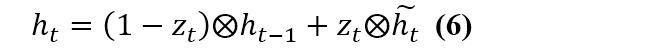



where the update gate and the reset gate are represented by *z_t_* and *r_t_*, respectively.The previous state is represented by *h_t_*_-1_, while the candidate state is represented by 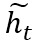 at time *t*. The sigmoid and hyperbolic tangent functions are represented by σ and *tanh*, respectively. *b_z_*, *b_h_*, and *b_r_* are all biases, whereas the operator 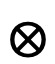 is element-wise multiplication.

#### LSTM Model

**Figure 5:**
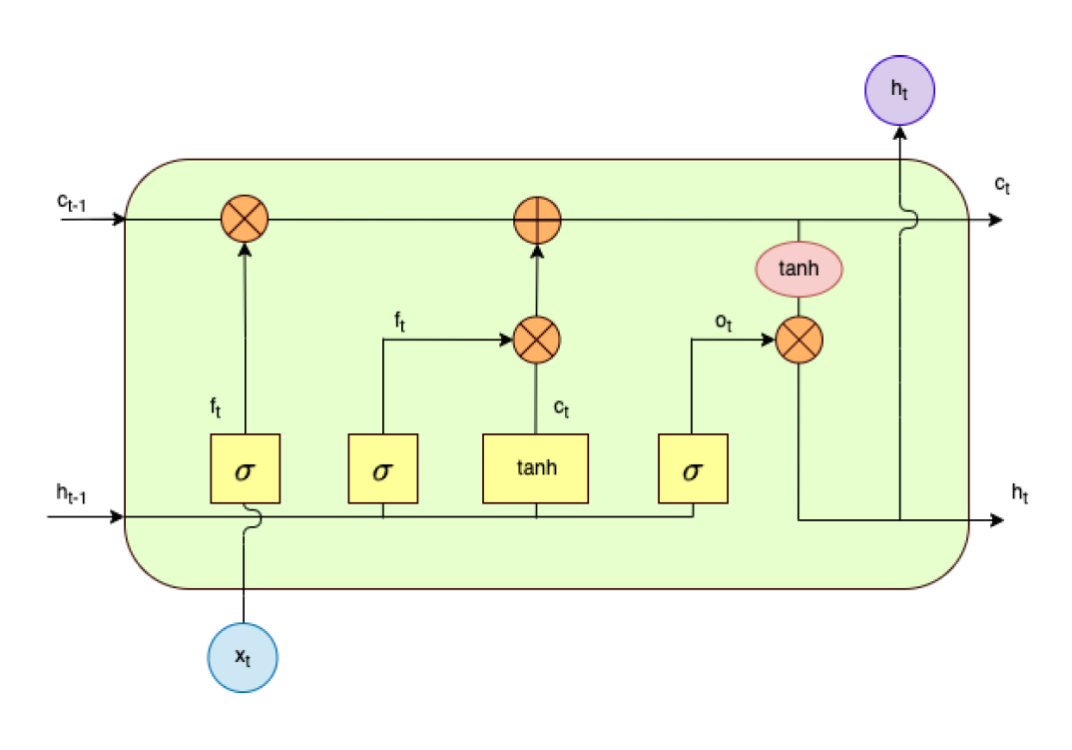
Long short-term memory model architecture: one of the recurrent neural network types. Its structure consists of input state (x_t_), hidden state (h_t_), output state (o_t_), forget gate (z_t_), and memory cell (c_t_).

LSTM is considered among the most widely adopted RNN types for sequential data-related tasks. It is designed to solve the problem of the traditional RNN model in capturing long-term dependencies, similar to GRU. The structure is shown in Figure 5.

Often, RNNs face the vanishing gradient problem when trying to capture long-term dependencies. Consequently, LSTM is designed to solve this issue by using some internal gates [44]. The LSTM architecture consists of 3 gates (input gate, output gate, and forget gate) along with a memory cell [45]. The goal of the memory cell is to store information over long sequences by capturing previous words (*c_t_*_-1_, *h_t_*_-1_), with some being dropped (forgotten) by the forget gate, which regulates the flow of information in (*x*) and out (*h*) of the cell by the 3 gates. The input gate, forget gate, output gate, and cell output can be illustrated as follows:

*f_t_* = *σ*(*W*^(^*^f^*^)^*x_t_* + *U*^(^*^f^*^)^*h_t_*_-1_ + *b*^(^*^f^*^)^) **(7)**

*i_t_* = *σ*(*W*^(^*^i^*^)^*x_t_* + *U*^(^*^i^*^)^*h_t_*_-1_ + *b*^(^*^i^*^)^) **(8)**

*o_t_* = *σ*(*W*^(^*^o^*^)^*x_t_* + *U*^(^*^o^*^)^*h_t_*_-1_ + *b*^(^*^o^*^)^) **(9)**

*c_t_* = *f_t_*

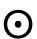

*c_t_*_-1_ + *i_t_*

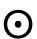

*tanh* (*W*^(^*^c^*^)^*x_t_* + *U*^(^*^c^*^)^*h_t_*_-1_ + *b*^(^*^c^*^)^) **(10)**

*h_t_* = *o_t_*

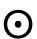

*tanh* (*c_t_*) **(11)**

where the input is represented by *x_t_* ∈ *R^d^* at time step *t*, while *d* is the feature dimension for each word. The element-wise sigmoid function and the element-wise dot product are represented by σ and 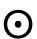, respectively. The memory cell represented by *c_t_* aims to solve the problem of vanishing gradients, which facilitates the learning of long-term dependencies. The forget gate, represented by *f_t_*, is used to decide what information to store in the memory cell, whereas the input gate and output gate, represented by *i_t_* and *o_t_*, respectively, aim to control the input and output of the memory cell, respectively. Finally, the output of LSTM is represented by *h_t_*.

**Figure 6:**
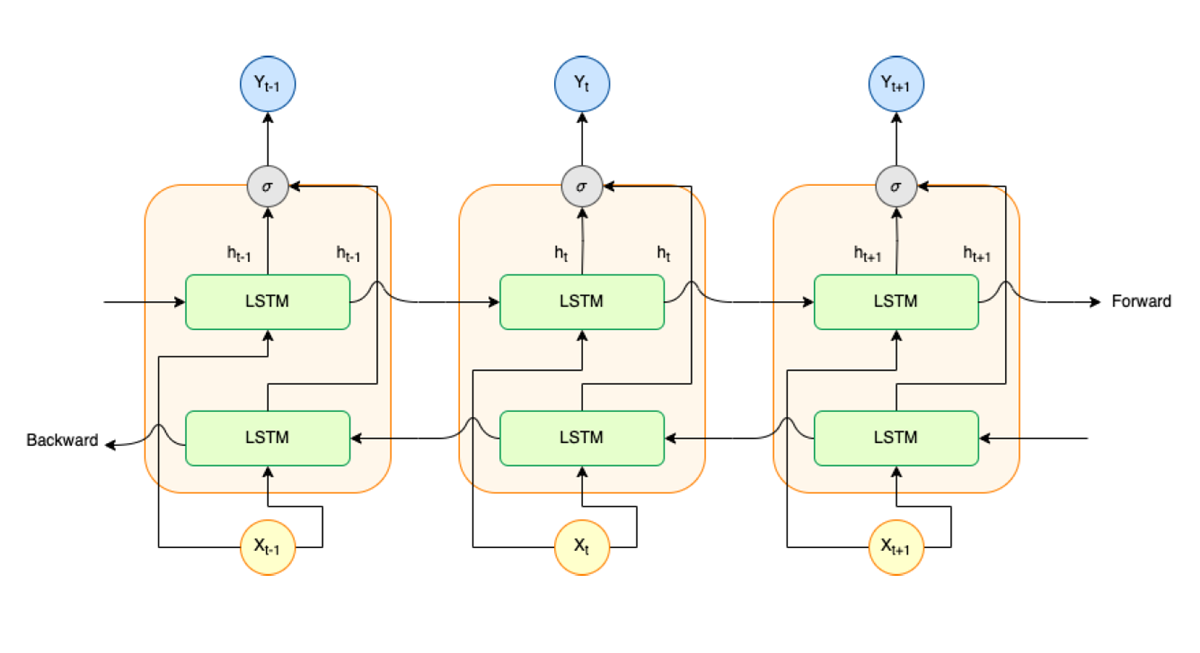
Bidirectional long short-term memory model architecture: one of the recurrent neural network types. Its structure consists of 2 long short-term memory parts: one processes input sequences in the forward direction, and the other processes input sequences in the backward direction. LSTM: long short-term memory.

However, LSTM still has some limitations, since it can only read in 1 direction (forward direction). In this case, the results may show low classification performance because some contextual information from future words may be ignored by considering just 1 forward direction. Thus, Alharbi et al [14] proposed a solution to address the limitations of LSTM, which is called BiLSTM. BiLSTM can capture long-term dependencies from both the past and the future of a sequence (2 directions), which is important for understanding the full context of the sequence. As an extension of LSTM, the BiLSTM network processes both the forward and backward directions of a sequence to capture dependencies [24,46,47]. The BiLSTM model architecture is illustrated in Figure 6.

#### CNN Model

**Figure 7:**
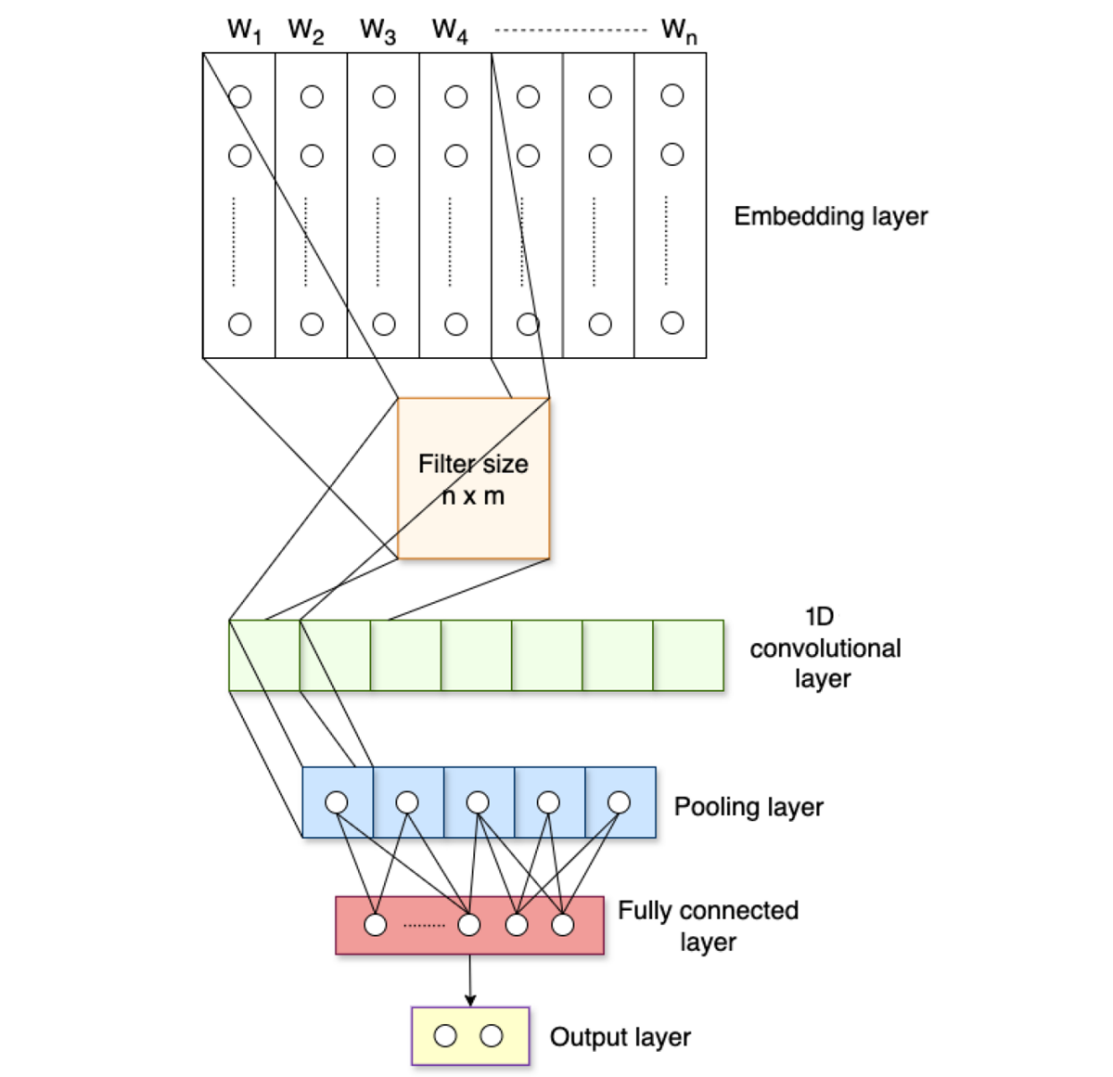
Convolutional neural network model architecture. Its structure consists of an embedding layer (input vectors), a 1D convolutional layer to extract local patterns, a pooling layer to select the most important features from the convolutional layer, a fully connected layer to prepare for the classification, and an output layer to calculate the output from the fully connected layer to the final binary classification result.

CNNs are generally developed for image classification. Currently, CNNs have shown strong performance in various NLP tasks, particularly in text classification. They are often considered appropriate for short text classification tasks [47-49]. A CNN model typically consists of several layers, which can be separated into 3 groups. First, the convolutional layer transforms the text data to obtain a feature map to detect the presence of a specific feature. Second, the pooling layer follows the convolutional layer, and it is designed to reduce the input data while preserving important information further. Third, the final stage of the CNN is the fully connected layer, which is responsible for using the features extracted in the previous layers for classification. The CNN architecture is illustrated in Figure 7.

Embedding layer: Let *w_i_* ∈ *R^m^* be a word vector with m-dimensional representation associated with a word in a sentence. A sentence of length *n* is represented as follows:

*w*_1:_*_n_* = *w*_1_
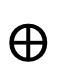
*w*_2_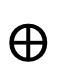 ...
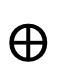
*w*_n_
**(12)**

where 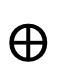 denotes the concatenation operator.

Convolutional layer: The filter is represented by *x* ∈ *R^h^*^×^*^m^*, where *h* is the number of words in a window and *m* is the dimension of a word vector. A feature map c = [*c*_1_, *c*_2_, …, *c_n_*_-_*_h_*_+1_] can be generated by:

*c_i_* = *f*(*x*∙*w_i_*_:_*_i_*_+_*_h_*_-1_ + *b*) **(13)**

where *b* ∈ *R* denotes a bias term and *f* is a nonlinear function.

Pooling layer: The pooling operation was applied for the respective filter to select the most important feature from each feature map. We used the max pooling operation to take the maximum value in the feature map.


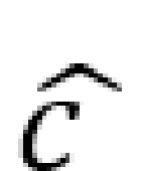
 = *max* {*c*} **(14)**

Fully connected layer: The selected features from the pooling layer are flattened into a single vector. It is passed to a fully connected layer to assign a probability for a specific label.



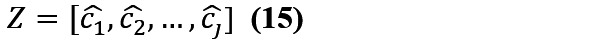



### Algorithmic Details

According to the deep learning models mentioned in the previous subsections, the key procedures can be described as follows: (1) import a dataset of reported symptoms of diseases (that are in Excel files) to our program; (2) perform data cleaning in sentences from Excel files; (3) create a vocabulary set with indexes from sentences to transform the sentences into vectors using a tokenizer; (4) create a word embedding from unique vocabulary for pretraining by using Word2Vec (skip-gram approach); (5) identify the longest vector with the maximum number of words in a sentence and adjust all the vectors to the same length as the longest (since the word count of words in a sentence can vary, the size of each vector can be different); (6) pad such vectors with fewer words than the maximum one with zeroes; (7) separate training data, validation data, and test data; (8) build the proposed models with BiLSTM, GRU, and CNN; (9) train the models according to the range given by the hyperparameters; and (10) evaluate the models.

We present the pseudocode of our methods, in which algorithm 1 was designed for BiLSTM and GRU (Textbox 1) and algorithm 2 was designed for CNN (Textbox 2). For CNN, we used a 1D convolution layer, and the feature map was selected by max pooling in convolutional features with kernel sizes of 4, 6, and 8. We used dropout and rectified linear unit (ReLU) as activation functions. Lastly, we used the Softmax activation function in a fully connected layer.

**Pseudocodes of bidirectional long short-term memory and gated recurrent unit for knee osteoarthritis predictions.**

Algorithm 1: Pseudocodes of bidirectional long short-term memory (BiLSTM) and gated recurrent unit (GRU)

Input: A collection of medical symptom reports for diseases in Excel files (Let *D* be one report and *S(i)* be an *i^th^* sentence: *D* = {*S*(1), *S*(2), *S*(3), … , *S*(*i*)}, *i* ∈ *N*)

Output: Prediction results/performance of BiLSTM and GRU

1: Import the reports of knee osteoarthritis data from Excel files into our program

2: while i in (1, n) do;

Select characters (that are Thai alphabets only) in the sentences

Tokenize by Word_Tokenize, DeepCut, AttaCut

Remove Thai stop words in sentences

Edit missing words with WOMAC (Western Ontario and McMaster Universities Osteoarthritis Index)-based processing approach (cutoff = 0.85)

end while

3: Create a set of vocabulary along with indexes by tokenizer from TensorFlow

4. Generate word embedding by Word2Vec (skip-gram approach)

vector_size = 128, epochs = 1000

5: Identify the max-length vector and adjust all vectors accordingly

6: Pad all smaller vectors with zeroes

7: Separate training, validation, and test datasets [training dataset: 80%, validation dataset: 10%, and test dataset: 10%]

8: Build the BiLSTM or GRU model

Set vocab_size, drop_out, BatchNomalization, and activation function parameters

9: while i <= epochs; // e = epoch numbers

execute the attention module;

import input vector;

Dropout; // (0.5)

BatchNormalization;

Update/optimize parameters with Adam optimization to minimize the loss function in the model

Verify the BiLSTM or GRU model using

validation data;

end while

10: Evaluate model by the test data;

Return the performance of BiLSTM and GRU

**Pseudocodes of convolutional neural network for knee osteoarthritis predictions.**

Algorithm 2: Pseudocodes of convolutional neural network (CNN)

Input: A collection of medical symptom reports for diseases in Excel files (Let *D* be one report and *S(i)* be an *i^th^* sentence: *D* = {*S*(1), *S*(2), *S*(3), … , *S*(*i*)}, *i* ∈ *N*)

Output: The result of the prediction from CNN

1: Import the report of knee osteoarthritis data

2: while i in (1, n) do;

Select characters (that are Thai alphabets only) in the sentences

Tokenize by Word_Tokenize, DeepCut, AttaCut

Remove Thai stop words in sentences

Edit missing words with the WOMAC (Western Ontario and McMaster Universities Osteoarthritis Index)-based processing approach (cutoff = 0.85)

end while

3: Create a set of vocabulary along with indexes by tokenizer from TensorFlow

4. Generate word embedding by Word2Vec (skip-gram approach)

vector_size = 128, epochs = 1000

5: Identify the max-length vector and adjust all vectors accordingly

6: Pad all smaller vectors with zeroes

7: Separate training, validation, and test datasets [training dataset: 80%, validation dataset: 10%, and test dataset: 10%]

8: Build the CNN model

Set vocab_size, drop_out, BatchNormalization, and activation function parameters

9: while i <= epochs; // e = epoch numbers

execute the attention module;

import input vector;

convolutional layer; // kernel_size = 4, 6, 8

Dropout; // (0.5)

Max pooling;

Fully connected layer;

Update/optimize parameters with Adam optimization to minimize the loss function in the model

Verify the CNN model using validation data;

end while

10: Evaluate model by using test data;

Return the performance of the CNN

### Evaluation Metrics

We evaluated our models by using 5 metrics: accuracy, sensitivity (or recall), specificity, precision, and *F*_1_-score. These parameters are defined as follows:

 Accuracy = (TP + TN) / (TP + FN + FP + TN) **(16)**

Sensitivity/recall = TP / (TP + FN) **(17)**

Specificity = TN / (TN + FP) **(18)**

Precision = TP / (TP + FP) **(19)**

*F*_1_-score = (2 × [precision × recall]) / (precision + recall) **(20)**

where true positive (TP) is the total number of patients classified and tagged in the dataset as OA, true negative (TN) is the total number of patients classified and tagged in the dataset as non-OA, false negative (FN) is the total number of patients classified as non-OA but tagged in the dataset as OA, and false positive (FP) is the total number of patients classified as OA but tagged in the dataset as non-OA. Accuracy is the ratio of true results of both OA and non-OA among the total number of patients examined. Sensitivity is the proportion of patients classified as OA by the model among all OA-tagged data. Specificity is the proportion of patients classified as non-OA by the model among all non-OA–tagged data. Precision is the percentage of patients classified as OA by the model, and *F*_1_-score is the average of recall and precision.

In summary, our contributions are as follows. First, our approach proposes to use clinicians’ or doctors’ notes (instead of x-ray or MRI images and statistical laboratory results), which contain meaningful messages from patients who have the same symptoms, to help in diagnosis. Second, we used the power of text classification through NLP in diagnosis, which can be used in complement with other diagnostic methods (such as using medical images or biospecimen laboratory results) to enhance accuracy and achieve a timely diagnosis. Third, we introduced the high potential of textual analysis based on doctors’ notes of symptoms to the field of knee OA as an alternative, which is new, and combined it with the WOMAC (dictionary/key terms for knee OA). Fourth, although our approach is performed on Thai datasets, the methodology can be adapted or repeated for diagnosing knee OA in other languages. Fifth, we combined and compared the text classification methods with deep learning models, including GRU, BiLSTM, and CNN, and at the end, we were able to identify the best combination in diagnosing knee OA in our approach. Sixth, to further tune our approach for better performance, we fine-tuned our method with our technique in adjusting misleading or wrong words by using real experts and physicians, as well as the Thai-verified version of the WOMAC.

## Results

### Records

To ensure data quality, we removed 888 (incomplete) records from 6737 knee OA records, leaving 5849 records containing complete patient reports. The dataset was separated into training, validation, and test sets comprising 4788, 598, and 463 records, respectively. We used the GRU, BiLSTM, and CNN models for knee OA prediction.

### Hyperparameters of GRU, BiLSTM, and CNN

The hyperparameters were adjusted for each model used in the experiments, including the quantity of neurons in each layer, the choice of the activation function, the optimization method, and the regularization techniques. In selecting hyperparameters for our experiments, we referred to relevant research studies in the literature that demonstrated strong performance [10,14,44,48,50]. The hyperparameters of each model are shown in Table 1.

**Table 1:** Hyperparameters of gated recurrent unit, bidirectional long short-term memory, and convolutional neural network.

Hyperparameter^a^	GRU^b^	BiLSTM^c^	CNN^d^
Filter	—^e^	—	32
Kernel	—	—	4, 6, 8
Activation function (last layer)	ReLU^f^ (Softmax)	ReLU (Softmax)	ReLU (Sigmoid)
Dropout	0.5	0.5	0.5
Epoch	50	50	50
Batch size	128	128	128
Loss function	categorical_crossentropy	categorical_crossentropy	binary_crossentropy
Optimizer/learning rate	Adam/0.0001	Adam/0.0001	Adam/0.0001
Regularization	Batch normalization	Batch normalization	Batch normalization

^a^BiLSTM: bidirectional long short-term memory.

^b^GRU: gated recurrent unit.

^c^CNN: convolutional neural network.

For GRU and BiLSTM, the weights were passed to the output layer with a Softmax function to diagnose knee OA. For CNN, the weights were passed to the output layer with a sigmoid function to diagnose knee OA. For all models, the hidden layer was trained and regularized using ReLU and batch normalization, respectively. To reduce the overfitting problem, we applied dropout [14], with a probability of 0.5 after each model while training with the Adam optimizer [10], using a learning rate of 0.0001 to optimize the model parameters. The experiments have two parts: (1) experiments that use the baseline approach and (2) experiments that use our WOMAC-based processing approach. In Tables 2 and 3, we present the experimental results of the models “before” processing the missing or edited words in sentences by using our WOMAC-based processing approach. The best-performing model was the BiLSTM model that used the tokenization of the AttaCut algorithm, with the following metrics: area under the curve (AUC), 0.85; accuracy, 0.87; precision, 0.85; sensitivity, 0.95; specificity, 0.76; and *F*_1_-score, 0.90.

In Tables 4 and 5, we present the experimental results of the models “after” processing the missing or edited words in sentences by using our WOMAC-based processing approach. The best-performing model was the BiLSTM model that used the tokenization of the AttaCut algorithm, with the following metrics: AUC, 0.91; accuracy, 0.91; precision, 0.91; sensitivity, 0.94; specificity, 0.87; and *F*_1_-score, 0.93.

**Table 2:** Results of the baseline approach (n=463) in terms of true positive, false positive, false negative, true negative, and runtime for each model.

Model			TP^a^, n (%)	FP^b^, n (%)	FN^c^, n (%)	TN^d^, n (%)	Runtime (s)
**Word_Tokenize (n=463)**							
	BiLSTM^e^		248 (53.6)	36 (7.8)	26 (5.6)	153 (33.0)	389.01
	GRU^f^		249 (53.8)	40 (8.6)	25 (5.4)	149 (32.2)	303.88
	CNN^g^		252 (54.4)	43 (9.3)	22 (4.8)	146 (31.5)	138.72
**DeepCut (n=463)**							
	BiLSTM		258 (55.7)	47 (10.2)	16 (3.5)	142 (30.7)	476.81
	GRU		249 (53.8)	46 (9.9)	25 (5.4)	143 (30.9)	411.99
	CNN		258 (55.7)	49 (10.6)	16 (3.5)	140 (30.2)	152.16
**AttaCut (n=463)**							
	BiLSTM		260 (56.2)	46 (9.9)	14 (3.0)	143 (30.9)	467.10
	GRU		251 (54.2)	40 (8.6)	23 (5.0)	149 (32.2)	387.65
	CNN		248 (53.6)	35 (7.6)	26 (5.6)	154 (33.3)	146.02

^a^TP: true positive.

^b^FP: false positive.

^c^FN: false negative.

^d^TN: true negative.

^e^BiLSTM: bidirectional long short-term memory.

^f^GRU: gated recurrent unit.

^g^CNN: convolutional neural network.

**Table 3:** Results of the baseline approach (n=463) in terms of accuracy, precision, sensitivity, specificity, and F1-score for each model.

Model		Accuracy (%)	Precision (%)	Sensitivity (%)	Specificity (%)	*F*_1_-score (%)
**Word_Tokenize**						
	BiLSTM^a^	0.86	0.85	0.92	0.77	0.89
	GRU^b^	0.86	0.86	0.91	0.79	0.89
	CNN^c^	0.87	0.87	0.91	0.91	0.89
**DeepCut**						
	BiLSTM	0.86	0.85	0.85	0.94	0.75
	GRU	0.85	0.84	0.83	0.91	0.84
	CNN	0.86	0.85	0.85	0.94	0.75
**AttaCut**						
	BiLSTM	0.87	0.85	0.95	0.76	0.90
	GRU	0.86	0.86	0.92	0.79	0.89
	CNN	0.87	0.88	0.91	0.81	0.89

^a^BiLSTM: bidirectional long short-term memory.

^b^GRU: gated recurrent unit.

^c^CNN: convolutional neural network.

**Table 4:** Results of the Western Ontario and McMaster Universities Osteoarthritis Index–based processing approach (n=463) in terms of true positive, false positive, false negative, true negative, and runtime for each model.

Model			TP^a^, n (%)	FP^b^, n (%)	FN^c^, n (%)	TN^d^, n (%)	Runtime (s)
**Word_Tokenize (n=463)**							
	BiLSTM^e^	252 (54.4)		24 (5.2)	22 (4.8)	165 (35.6)	359.62
	GRU^f^	256 (55.3)		36 (7.8)	18 (3.9)	153 (33.0)	294.49
	CNN^g^	254 (54.9)		25 (5.4)	20 (4.3)	164 (35.4)	128.76
**DeepCut (n=463)**							
	BiLSTM	251 (54.2)		25 (5.4)	23 (5.0)	164 (35.4)	429.84
	GRU	250 (54.0)		27 (5.8)	24 (5.2)	162 (35.0)	367.58
	CNN	258 (55.7)		36 (7.8)	16 (3.5)	153 (33.0)	129.69
**AttaCut (n=463)**							
	BiLSTM	257 (55.5)		24 (5.2)	17 (3.7)	165 (35.6)	397.51
	GRU	254 (54.9)		31 (6.7)	20 (4.3)	158 (34.1)	343.44
	CNN	251 (54.2)		22 (4.8)	23 (5.0)	167 (36.1)	117.44

^a^TP: true positive.

^b^FP: false positive.

^c^FN: false negative.

^d^TN: true negative.

^e^BiLSTM: bidirectional long short-term memory.

^f^GRU: gated recurrent unit.

^g^CNN: convolutional neural network.

**Table 5:** Results of the Western Ontario and McMaster Universities Osteoarthritis Index–based processing approach (n=463) in terms of accuracy, precision, sensitivity, specificity, and F1-score for each model.

Model		Accuracy (%)	Precision (%)	Sensitivity (%)	Specificity (%)	*F*_1_-score (%)
**Word_Tokenize**						
	BiLSTM^a^	0.90	0.91	0.93	0.87	0.92
	GRU^b^	0.88	0.88	0.93	0.81	0.90
	CNN^c^	0.90	0.91	0.92	0.87	0.92
**DeepCut**						
	BiLSTM	0.89	0.88	0.94	0.81	0.91
	GRU	0.89	0.90	0.91	0.86	0.91
	CNN	0.90	0.91	0.92	0.87	0.91
**AttaCut**						
	BiLSTM	0.91	0.91	0.94	0.87	0.93
	GRU	0.89	0.89	0.93	0.84	0.91
	CNN	0.90	0.92	0.92	0.88	0.92

^a^BiLSTM: bidirectional long short-term memory.

^b^GRU: gated recurrent unit.

^c^CNN: convolutional neural network.

## Discussion

### Overview

While conventional medical data, such as images and statistical or laboratory data, have been extensively used, the use of textual data through NLP for knee OA diagnosis is still a relatively new approach. As mentioned previously, we propose an NLP model to detect the occurrence of knee OA by incorporating deep learning models, including GRU, BiLSTM, and CNN, based on textual data of knee OA symptoms reported in doctors’ notes. We discuss principal findings, comparison with prior work, strengths, limitations, and future work in the following subsections.

### Principal Findings

A key finding in this study is that our method, using only textual data from doctors’ notes for knee OA prediction, performed better than previous studies that used image data and statistical or laboratory data. Moreover, in our experiments, the models trained very quickly due to the nature of our textual data (short sentences), which, in turn, can reduce the model’s runtime. We also found that the best tokenization algorithm and model for this experiment was BiLSTM combined with AttaCut tokenization.

Although CNNs can extract relevant local features in sentences where specific combinations of words (local features) are important, it was not designed to handle sequential data like our data. Therefore, CNNs may not effectively handle variable-length sequences. GRUs have a simpler architecture and train faster than BiLSTM. However, GRUs may not perform as well as BiLSTM on very complex sequence data. This may be because BiLSTM can address the issues of complex sequences or long-distance dependencies in sentences better, as shown in our experiments, and thus, BiLSTM had the best performance. The best-performing model “before” applying our WOMAC-based processing approach was BiLSTM (AUC, 0.85; accuracy, 0.87; precision, 0.85; sensitivity, 0.95; specificity, 0.76; *F*_1_-score, 0.90), and after applying our approach, there was an improvement in the results with BiLSTM (AUC, 0.91; accuracy, 0.91; precision, 0.91; sensitivity, 0.94; specificity, 0.87; *F*_1_-score, 0.93). Therefore, our WOMAC-based processing approach can increase the performance of models. A comparison of the baseline model and the model using the WOMAC-based processing approach is shown in Table 6.

**Table 6:** Comparison between the baseline approach and Western Ontario and McMaster Universities Osteoarthritis Index–based processing approach, and the improvement rate from baseline for each model.

Model			Baseline							WOMAC^a^-based processing approach							Improvement rate					
			*F*_1_-score (%)		Macro avg^b^ (%)		Weighted avg^c^ (%)		*F*_1_-score (%)			Macro avg^b^ (%)		Weighted avg^c^ (%)		*F*_1_-score (%)			Macro avg^b^ (%)		Weighted avg^c^ (%)	
**Word_Tokenize**																						
	BiLSTM^d^	0.89		0.88		0.89		0.92			0.90		0.90		0.03			0.02		0.01		
	GRU^e^	0.88		0.85		0.86		0.90			0.88		0.88		0.02			0.03		0.02		
	CNN^f^	0.89		0.86		0.87		0.92			0.90		0.90		0.03			0.04		0.03		
**DeepCut**																						
	BiLSTM	0.89		0.85		0.86		0.91			0.88		0.89		0.02			0.03		0.03		
	GRU	0.88		0.84		0.85		0.91			0.89		0.89		0.03			0.04		0.04		
	CNN	0.89		0.85		0.86		0.91			0.89		0.90		0.02			0.04		0.04		
**AttaCut**																						
	BiLSTM	0.90		0.86		0.87		0.93			0.91		0.91		0.03			0.05		0.04		
	GRU	0.89		0.86		0.86		0.91			0.88		0.89		0.02			0.02		0.03		
	CNN	0.89		0.86		0.87		0.92			0.88		0.89		0.03			0.02		0.02		

^a^AUC: area under the curve.

^b^MAE: mean absolute error.

^c^Not applicable.

### Comparison With Prior Work

In comparisons of our study with prior studies, we considered the primary data source used (image data or statistical data). Sources of data are presented in Multimedia Appendix 4. A detailed comparison of the model in our study with the models in previous studies is shown in Table 7. When compared to prior models that used image data [1,17,22], our model showed better accuracy [1,17] and a higher AUC [22]. When compared to prior models that used statistical data [2,6,19], our model showed higher accuracy, sensitivity, specificity, and AUC [2,6]. However, the AUC of our model was lower than that of the model in the study by Halilaj et al [19].

**Table 7:** Comparison of our model with models in previous studies.

Study (data source)	Number of patients	Accuracy (%)	AUC^a^ (%)	MAE^b^ (%)	Precision (%)	Sensitivity (%)	Specificity (%)	*F*_1_-score (%)
Brahim et al [1] (images)	1024	0.83	—^c^	—	—	0.87	0.81	—
Shen et al [2] (stats)	195	—	—	0.11	—	0.71	0.67	0.69
Halilaj et al [19] (stats)	3924	—	0.95	—	—	—	—	—
Yong et al [17] (images)	4796	0.88	—	0.33	—	—	—	—
Lim et al [6] (stats)	5749	0.72	0.77	—	—	0.67	0.73	—
Tiulpin et al [22] (images)	4928	—	0.79	—	—	—	—	—
Our study	5849	0.91	0.91	0.09	0.91	0.94	0.87	0.93

^a^AUC: area under the curve.

^b^MAE: mean absolute error.

^c^Not applicable.

### Strengths

The overall performance of our model surpasses that of models in previous studies. Our proposed method introduces a novel approach to knee OA prediction research. Our research has 3 strengths. First, our model uses textual data, a valuable information source for AI learning. While textual data is critical, investigation and verification steps are also important. Second, since the nature of our dataset is textual and typically consists of short sentences, it does not require much processing time; hence, our model can be trained very quickly. Third, our patient dataset is larger than the datasets in previous studies (Table 7).

### Limitations

A limitation of our method is that it currently works only with the Thai language. As a result, it requires local application programming interfaces (APIs) that can handle the Thai language for processing. However, these local APIs may not have a programmer community as large as that for English or global APIs, and therefore, they may not be as productive. Our work focused on using textual data only in our model, and thus, we did not incorporate other types of data, such as image data and statistical or laboratory data, into our model, which could have improved diagnostic performance.

### Future Work

In the future, we aim to improve our proposed method by incorporating image data and statistical data to achieve even better knee OA prediction. Additionally, we want to extend our work to make the model more precise by predicting the stage of knee OA (there are 5 stages based on WOMAC scores, ranging from 0 to 4) [12,16,18]. Furthermore, our proposed method can be applied to other medical conditions or diseases, such as depression.

### Conclusions

We proposed a method to predict the occurrence of knee OA by using clinicians’ or doctors’ notes. This method has better prediction performance than other conventional methods that use images or statistical laboratory data. We confirmed the possibility of using symptom texts reported by patients (recorded by doctors) to predict knee OA conditions. Moreover, we demonstrated that the text of symptom reports can be a valuable data source to predict or classify a disease (whether a particular knee will have OA progression or not). Furthermore, our method involving text data (mostly consisting of short sentences) can save a considerable amount of time compared with other methods that use image data and statistical laboratory data (patients must visit the hospital and undergo imaging or tests to generate such data).

## Appendix

Multimedia Appendix 1 CONSORT (Consolidated Standards of Reporting Trials) checklist.

Multimedia Appendix 2 CONSORT-EHEALTH (Consolidated Standards of Reporting Trials of Electronic and Mobile Health Applications and Online Telehealth) checklist.

Multimedia Appendix 3 STARD (Standards for Reporting of Diagnostic Accuracy Studies) checklist.

Multimedia Appendix 4 Sources of data used according to the literature.

## Abbreviations

AI: artificial intelligence
API: application programming interface
AUC: area under the curve
BiLSTM: bidirectional long short-term memory
CBOW: continuous bag of words
CNN: convolutional neural network
GRU: gated recurrent unit
KL: Kellgren/Lawrence
LSTM: long short-term memory
MRI: magnetic resonance imaging
NLP: natural language processing
OA: osteoarthritis
ReLU: rectified linear unit
RNN: recurrent neural network
WOMAC: Western Ontario and McMaster Universities Arthritis Index

## Data Availability

The datasets used in this study are not publicly available, as they require institutional approval on a case-by-case basis from the relevant authorities. However, the contact information of the relevant authorities can be requested by contacting the corresponding author.
