# Supplementary figures and images for: Early Diagnosis of Knee Osteoarthritis With a Natural Language Processing–Driven Approach Based on Clinician Notes: Development and Validation Study
Source: JMIR Form Res. 2025 Aug 14;9:e64536. doi: 10.2196/64536 (PMC12395113; doi:10.2196/64536)

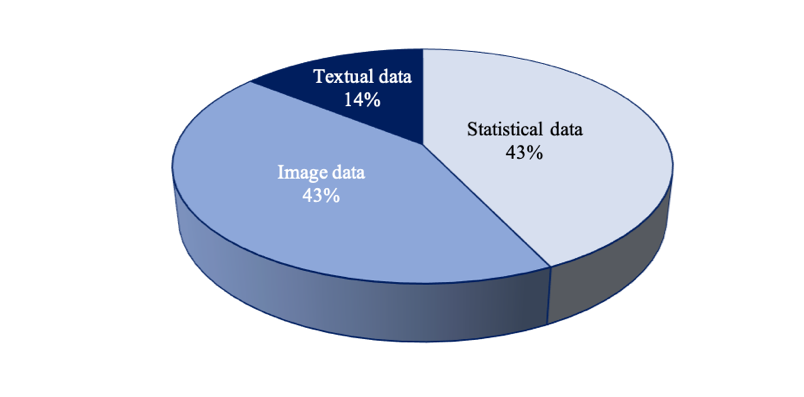

Supplement: Multimedia Appendix 4 [file formative_v9i1e64536_app4.png]
